# TRAJECTORY OF DECLINING PHYSICAL FUNCTION AMONG NURSING HOME RESIDENTS IN JAPAN

**DOI:** 10.1093/geroni/igae098.3175

**Published:** 2024-12-31

**Authors:** Kasumi Ikuta, Maiko Noguchi-Watanabe, Miya Aishima, Sakiko Fukui

**Affiliations:** Tokyo Medical and Dental University, Bunkyo-ku, Tokyo, Japan; Tokyo Medical and Dental University, Bunkyo-ku, Tokyo, Japan; Tokyo Medical and Dental University, Bunkyo-ku, Tokyo, Japan; Tokyo Medical and Dental University, Bunkyo-ku, Tokyo, Japan

## Abstract

The timing and speed of physical function decline among nursing home residents vary, and the decline trajectory remains unclear. Therefore, this multicenter retrospective study was aimed at identifying the trajectory of declining physical function among Japanese nursing home residents. We included new nursing home residents who were admitted between July 2021 and March 2023, who had a high physical function score (Barthel Index [BI]: >60), and whose physical function declined during the period from admission to 6 months after. We evaluated the BI monthly as an indicator of the residents’ physical function status. We conducted a group-based trajectory model analysis to find the physical functional decline. Four types of models were identified. Furthermore, we compared the trajectory model to other trajectory models using the Akaike Information Criterion. Based on the results, we included 163 new residents, including 106 women (65.0%), with a mean age of 86.16 years (SD = 5.84). We selected the five groups trajectory model: slight decline, slightly larger decline, maintenance after decline, decline after maintenance, and large decline. The characteristics of the large-decline group were a diagnosis of dementia and low motivation and those of the maintenance-after-decline group were female sex and low body mass index and oral function. We identified the trajectory group based on the timing and speed of physical function decline. We believe that nursing home staff assess the trajectories of physical function decline and individualize residents’ interventions accordingly.

